# Intrathyroidal parathyroid carcinoma presenting as thyroid nodule: Case report of unusual location

**DOI:** 10.1016/j.amsu.2022.103994

**Published:** 2022-06-15

**Authors:** Anajar Said, Allaoui Abire, Laidi Soukaina, Alatawna Hind, Taali Loubna, Tahiri Ilias, Hajjij Amal, Saadi Mustapha, Zalakh Mohammed, Benariba Fouad

**Affiliations:** a ENT Department, Face and Neck Surgery, Hospital Cheikh Khalifa, Mohammed VI University of Health Sciences, Casablanca, Morocco; b internal Medecin Department, Hospital Cheikh Khalifa, Mohammed VI University of Health Sciences, Casablanca, Morocco; c Endocrinology Department, Hospital Cheikh Khalifa, Mohammed VI University of Health Sciences, Casablanca, Morocco; d Pathologist-Anatomical, IBN Zohr Center, Casablanca, Morocco; e ENT Department, Face and Neck Surgery, Military Hospital Mohammed V, Rabat, Morocco

**Keywords:** Intra thyroidal parathyroid carcinoma, Immunohistochemistry, Thyroidectomy

## Abstract

**Introduction:**

Parathyroid carcinoma (PC) is considered a rare and uncommon malignancy. Its prevalence is about 0.005% of all cancers. Intrathyroidal location is rare, rendering preoperative diagnosis tedious. Until now, around 700 cases of PC have been documented, reportedly, among them, less than 21 cases of intrathyroidal parathyroid carcinoma have been described in the literature. We report a case of intrathyroidal PC that was taken for a suspicious thyroid nodule, with a literature review.

PRESENTATION OF THE CASE: Our case is an asymptomatic intrathyroidal PC imitating a suspicious thyroid nodule, in a 54-year-old woman. A literature review was performed about clinical, radiological features, histopathological findings, and therapeutic options.

**Discussion:**

The diagnosis of asymptomatic intrathyroidal parathyroid carcinoma, similar to our case report, is even more difficult, our patient had no symptoms of hypercalcemia. Surgery is the cornerstone of the treatment. A better chance to cure this disease is conditioned by complete surgical resection with negative margins microscopically, that was the case of our patient with a very good clinical course after 12 months of follow-up.

## Highlights

• Parathyroid Carcinoma Is an Uncommon Endocrine Malignancy, Making Approximately 0.005% of All Cancers and 0,5%–2% of Patient With Primary Hyperparathyroidism.
• Intrathyroidal Location Is Rare and Make Preoperative Suspicion and Diagnosis Challenging.
• Approximately 700 Cases of Parathyroid Carcinoma Have Been Reported to Date and, to The Best of Our Knowledge, Less Than 24 Cases of Intrathyroidal Parathyroid Carcinoma Has Been Previously Documented.

## 1 Introduction

PC represents less than 1% of all cases of primary hyperparathyroidism (PHPT) [1]. Parathyroid carcinoma can have an ectopic location in very rare occasions caused by inadequate migration of parathyroid gland(s) during embryogenesis [2]. A better chance to cure this disease is conditioned by a complete surgical resection with negative margins microscopically, which is the primary treatment [3], but no universal consensus was produced concerning management and follow-up.

We present here an unusual, asymptomatic case of parathyroid carcinoma emerging from intrathyroidal parathyroid tissue, with a misleading primary diagnosis of a suspicious thyroid nodule. It adds to the limited data on the subject, after a thorough literature review, only 21 similar cases have been found [1]. The work has been reported in line with the SCARE 2020 criteria [10].

## 2 Case report

A 54-year-old woman, without a personal or a family history, has presented to our institution for a swelling on the median part of the neck, evolving during six years, the swelling had increased in size over that period of time. There was no history of systemic or local infection, trauma or surgical intervention. No dysphagia, no shortness of breath, or hoarseness were noted.

**Fig. 1:**
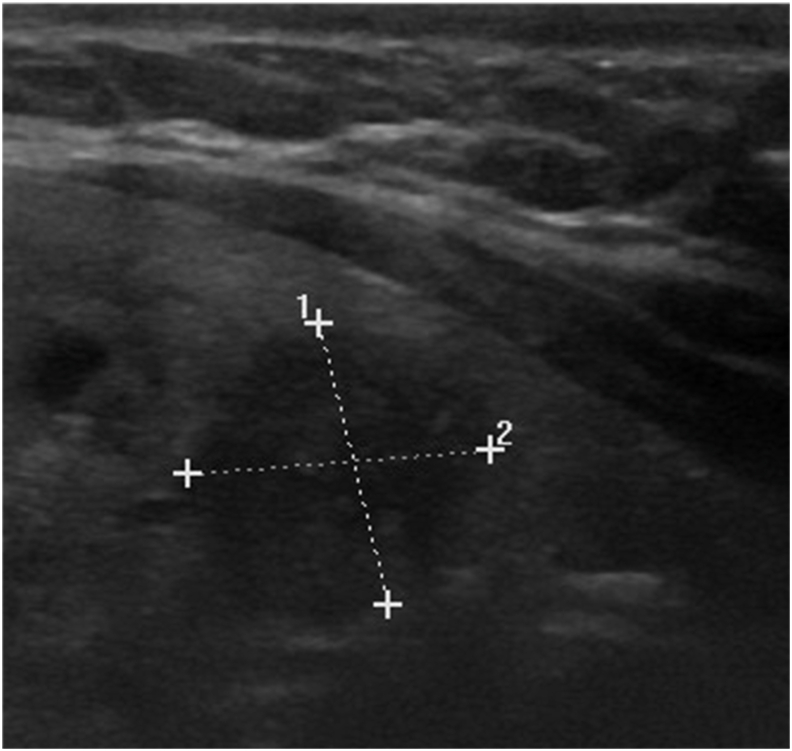
Ultrasounds showed a hypoechoic and plunging nodule tirads 5, 35*25MM.

She did not complain of hypercalcemia symptoms such as weakness, bone pain, or polyuria. Clinical examination revealed thyroid nodules, and no lymph nodes were detected. Laboratory exam found normal level of TSH. Ultrasounds also showed a hypoechoic nodule EU TIRADS 5, sized 30*20mm with multiple calcifications at left, and another nodule EU TIRADS 3 at right (Fig. 1). Surgery was performed in order to solve her thyroid problem. The operation was undergone without any complications. The patient left the hospital after 48h.

**Fig. 2:**
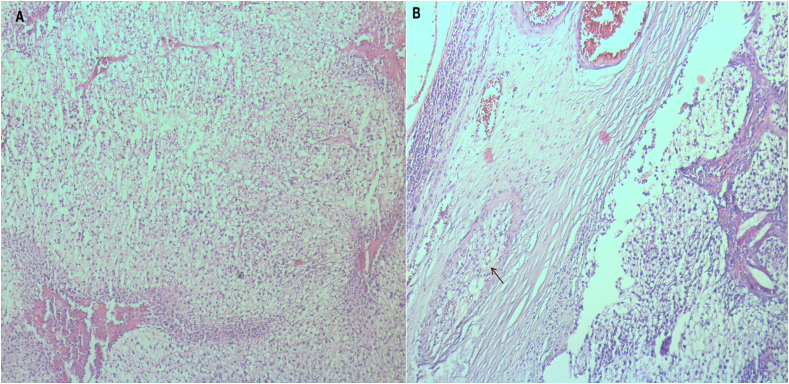
Histopathology; A: Malignant proliferation of clear cells B: Vascular invasion (arrow).

**Fig. 3:**
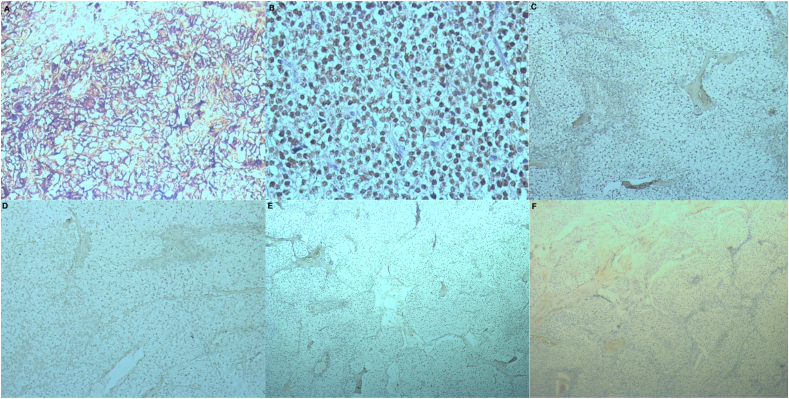
Immunohistochemistry: A. Immunohistochemistry (GX20) antichromogranin antibody: Positive. B. Immunohistochemistry (GX20) anti GATA3 antibody: Positive. C. Immunohistochemistry (GX10) anti P63 antibody: Negative. D. Immunohistochemistry (GX10) antithyroglobulin antibody: Negative. E. Immunohistochemistry (GX10) anti TTF1 antibody: NEGATIVE. F. Immunohistochemistry (GX5) anticalcitonine: Negative.

Histopathology of the left nodule revealed a malignant proliferation of clear cells with necrosis and vascular invasion (Fig. 2). On the immunohistochemistry, tumor cells were negative for thyroid transcription factor 1 (TTF-1), thyroglobulin, calcitonin antibodies (Fig. 3) excluding a thyroid carcinoma especially medullary subtype. Neoplastic cells were positive to GATA3 antibody (Fig. 3), it is a transcription factor involved in the embryonic development of the parathyroid glands.Chromogranin A antibody was positive (Fig. 3). It is also stocked in the parathyroid cells. The clinical pathological confrontation and the results of the immunohistochemistry study have us conclude to the diagnosis of a clear cell parathyroid carcinoma.Blood workup showed increased serum intact parathyroid hormone level of 230 pg/mL (normal values are 10–55 pg/mL) Levels of serum phosphorus and calcium were normal. After 12 months, no metastases or local recurrence, or were observed.

## 3 Discussion

Intrathyroidal PC is the most uncommon site of an ectopic parathyroid gland, accounting for 0.2% [3,4]. Until now, around 700 cases of parathyroid carcinoma have been documented, reportedly, in the literature less than 21 cases of have been described [5,6]. No definitive risk factors have been found [1,4]. Commonly, patients with parathyroid cancer presents with symptoms depicting a severe hyperparathyroidism and hypercalcemia.

Usually, a neck mass can be palpable in 30%–75% of cases [1].

Our case was unusual, because it was asymptomatic on presentation, apart from the thyroid nodule.

The diagnosis remains challenging. Blood workup (serum calcium, intact parathyroid hormone) and ultrasonography are very helpful. The localization of the affected parathyroid gland has been eased using MRI of the cervical area and Sestamibi scan, but it remains difficult to differentiate between adenoma and carcinoma, using these exams. The same thing was described for fine needle aspiration, there is a huge resemblance in cytological features of thyroid nodules and parathyroid nodules [7].

The pathogenesis of parathyroid cancer remains obscure [11]. Surgery is the corner stone of the cure. The standard of treatment is a surgery resection with microscopically negative margins, it is considered to be the best treatment in this situation [7].

This technique involves a tumor resection associated to ipsilateral thyroid lobectomy and cervical lymphadenectomy. All the concerned structures or local metastatic lymph nodes should be removed [8].

Postoperative blood testing of PTH level and serum calcium is the way to evaluate the efficacy of the treatment. If complete resection is achieved, the survival rate is approximately 90%, if not, the risk of a local recurrence is about 50%–60% [8]. The efficacy of radiotherapy in local or metastatic disease as a primary therapy is not proved [9].

## 4 Conclusion

Parathyroid carcinoma is already a rare entity, parathyroid carcinoma emerging in an ectopic location, especially the intrathyroidal site, is even more scarce. When detected outside its typical sites, it is particularly challenging to be diagnosed, especially in asymptomatic patients, and can be taken for a thyroid nodule. Complete surgical resection with microscopically clean margins is the golden standard and allows the best chance of cure.

## Ethical approval

Written informed consent was obtained from the patient for publication of this case report and accompanying image.

## Sources of funding

No sources of funding to declare.

## Authors' contribution

Said Anajar: Corresponding author.

Amal Hajjij, Laidi Soukaina: writing the paper.

Fouad Benariba: study concept.

## Trial registry number

Not “First Man “studies.

## Guarantor

ANAJAR SAID.

## Consent

Written informed consent was obtained from the patient for publication of this case report and accompanying image.

## Provenance and peer review

Not commissioned, externally peer reviewed.

## Declaration of competing interest

All the authors have no personal or financial conflicts of interest regard this case report.
